# Immune recruitment or suppression by glycan engineering of endogenous and therapeutic antibodies^☆^

**DOI:** 10.1016/j.bbagen.2016.04.016

**Published:** 2016-08

**Authors:** Ngoc Phuong Lan Le, Thomas A. Bowden, Weston B. Struwe, Max Crispin

**Affiliations:** a Oxford Glycobiology Institute, Department of Biochemistry, University of Oxford, South Parks Road, Oxford OX1 3QU, United Kingdom; b Division of Structural Biology, University of Oxford, Wellcome Trust Centre for Human Genetics, Roosevelt Drive, Oxford OX3 7BN, United Kingdom

**Keywords:** Antibody, Glycosylation, Structure, Therapeutic antibodies, Effector function, Glycan

## Abstract

Human serum IgG contains multiple glycoforms which exhibit a range of binding properties to effector molecules such as cellular Fc receptors. Emerging knowledge of how the Fc glycans contribute to the antibody structure and effector functions has opened new avenues for the exploitation of defined antibody glycoforms in the treatment of diseases. Here, we review the structure and activity of antibody glycoforms and highlight developments in antibody glycoengineering by both the manipulation of the cellular glycosylation machinery and by chemoenzymatic synthesis. We discuss wide ranging applications of antibody glycoengineering in the treatment of cancer, autoimmunity and inflammation. This article is part of a Special Issue entitled "Glycans in personalised medicine" Guest Editor: Professor Gordan Lauc.

## Highlights

• IgG glycosylation impacts antibody effector function.
• Modulation of antibody glycosylation has therapeutic potential.
• Antibody glycoforms exhibit anti- and pro-inflammatory properties.

## 1 Introduction

Antibodies, also known as immunoglobulins (Ig), are naturally produced by plasma cells derived from B cells and play a key role in humoral immunity. Human antibodies are classified into five isotypes (IgA, IgD, IgE, IgG and IgM) each with distinct structure and biological activity and the heavy chain within each class are designated α, δ, ε, γ and μ, respectively [1]. Among these five antibody isotypes, IgG has the longest serum half-life, is the most abundant (~ 75%) in circulation [2] and is the only isotype used in licensed recombinant monoclonal antibody therapeutics. These recombinant therapies have mainly utilized the IgG1 subclass [3] and have been developed to treat a large range of diseases such as cancer, autoimmunity and inflammation.

**Fig. 1:**
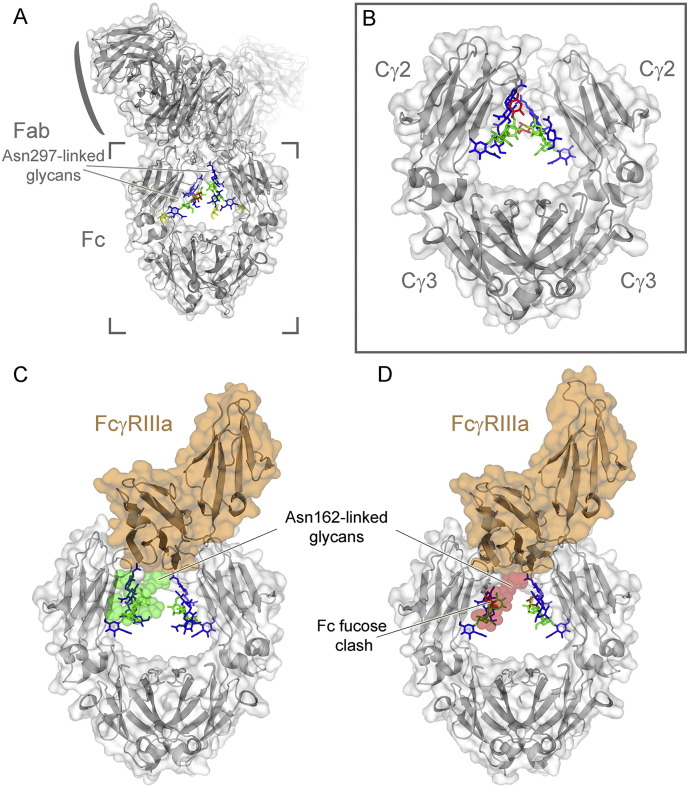
Overall architecture of IgG, the IgG Fc domain, and the interaction with FcγRIIIa. (A) Crystal structure of an intact human IgG1 with the protein moiety shown in gray and the glycans as sticks. Monosaccharide residues are coloured: mannose (green), fucose (red). galactose (yellow), N-acetylglucosamine (blue). PDB ID: 1HZH[281], [282]. (B) Crystal structure of an isolated fucosylated IgG1 Fc domain (PDB ID: 3AVE) [47]. (C and D) Fucosylation of IgG Fc glycan impairs FcγRIIIA binding through a steric clash with the Asn162 glycan of the receptor (transparent surface; green, no clash; red, clash with Fc fucose).

One promising strategy to improve the efficacy of therapeutic antibodies is manipulation of antibody glycosylation [4]. Normal serum human IgG contains multiple glycoforms due to the addition of diverse complex biantennary oligosaccharides in the IgG Fc domain. Although the IgG Fc glycans do not affect antigen binding by the antibody Fab domains, they are essential to the IgG effector functions. For example, antibody-dependent cellular cytotoxicity (ADCC) can be greatly enhanced by removal of the core fucose residue from the IgG Fc glycan [5], [6], [7], [8] (Fig. 1). Conversely, the addition of terminal sialic acid residues to the IgG Fc glycan has been reported to suppress autoantibody-driven inflammation [9], [10], [11], [12]. In this review, we discuss the structure and activity of different IgG antibody glycoforms and highlight the emerging strategies of antibody glycoengineering to augment biotherapeutic attributes. Overall, we outline the current knowledge of structure-based IgG glycoform engineering.

## 2 Overview of Fc structure and effector functions

The monomeric form of IgG represents the prototypical antibody structure. It has an overall Y-shaped structure with two ~ 50 kDa heavy chains and two ~ 25 kDa light chains, each of which contains a variable (V) and a constant (C) region [13], [14]. The heavy (γ) chains of IgG consist of four Ig domains, V_H_, Cγ1, Cγ2 and Cγ3, the light (either κ- or λ-type) chains consist of two Ig domains, V_L_ and C_L_. Together, the heavy and light chains form three distinctive features of an antibody: the antigen-binding domain F(ab^′^)_2_, the flexible hinge region, and the Fc domain (Fig. 1A). The Fc domain promotes effector functions via interactions with the complement system and the cellular Fcγ receptors (FcγRs) present on various immune cells [15].

Human IgGs can be further divided into four subclasses, IgG1, IgG2, IgG3 and IgG4, which are named in order of decreasing abundance [16]. Different subclasses can extend different effector functions. For example, IgG1 and IgG3 trigger the classical route of complement more efficiently than IgG2 and IgG4 [17], [18], [19]. Another major distinction is that IgG1 and IgG3 exhibit higher affinity to most FcγRs than IgG2 and IgG4 [20]. Between IgG1 and IgG3, the affinity of IgG3 to most FcγRs tends to exceed that of IgG1 [20]. The four human IgG subclasses sequences are relatively conserved. However, the length and flexibility of their hinge regions vary extensively with IgG3 [62 amino acids (aa)] > IgG1 (15 aa) > IgG2 = IgG4 (12 aa); yet, in terms of the hinge flexibility, the order is: IgG3 > IgG1 > IgG4 > IgG2 [21], [22]. This variation in flexibility is likely impacted by the variable number of interchain disulphides across the antibody classes.

The IgG1 Fc domain mediates antibody effector functions and is structured as a horseshoe-like homodimer comprising of the Cγ2 and Cγ3 domains that are linked together by two interchain disulphide bonds at the lower hinge [23]. The Fc domain contains various binding sites for several host and bacterial proteins as well as the human cytosolic Fc binding protein, TRIM21, that relocates IgG bound virion complexes to the proteasome for degradation [24]. While the FcγRs and complement molecule C1q interact with the Cγ2 domain and the lower hinge region, bacterial staphylococcal protein A and the neonatal Fc receptor (FcRn) bind to the interface of Cγ2 and Cγ3 domains [25], [26], [27]. The two opposing Cγ3 domains form extensive hydrophobic interactions that involve more than 20 residues per chain. In contrast, the interaction between the opposing and flexible Cγ2 domains is principally through the conserved N-linked glycan attached at Asn297, which projects along the surface of the Cγ2 domains and occupies the interstitial space between domains [23]. The changes in naturally occurring and engineered Fc glycosylation and how they affect IgG structure and effector functions will be discussed in more detail in subsequent sections.

## 3 Antibody glycoforms

### 3.1 Biogenesis and typical serum composition of human antibody glycoforms

Antibody N-linked glycans are assembled via the conserved endoplasmic reticulum (ER) and Golgi glycosylation pathway. Interestingly, Fc glycosylation largely remains consistent when isolated from human serum as well as from different recombinant expression systems. The glycans are not homogeneous but do not exhibit the very significant heterogeneity, often characteristic of other glycoproteins with more solvent accessible glycans, due to extensive intramolecular glycan-protein interaction within the Fc.

Like all glycoproteins, IgG N-linked glycans are added co-translationally in the ER to the amide nitrogen of the asparagine (Asn) side chain within the glycosylation sequon (Asn-X-Ser/Thr, where X is any amino acid except Pro). Glycosylation is initiated by the *en bloc* transfer of Glc_3_Man_9_GlcNAc_2_ from a Glc_3_Man_9_GlcNAc_2_-pyrophosphate-dolichol precursor by oligosaccharyltransferase to a nascent polypeptide in the lumen of the ER. The glucosylated N-glycan structure is then modified by a series of glycosidases and glycosyltransferases during transit through the secretory system. After protein folding and initial trimming by the ER-resident glucosidases I and II and α-mannosidase, the nascent antibody is released from the calnexin–calreticulin quality control checkpoint and enters the Golgi apparatus where further glycan processing occurs. Glycan processing in the Golgi combines both glycan trimming, catalysed by mannosidases I and II, as well as stepwise addition of individual monosaccharide residues, catalysed by GlcNAc-transferases I, II and III (GnT I, II and III), fucosyltransferases (FucT), galactosyltransferases (GalT) and sialyltransferases (SiaT), leading to a diversity of IgG glycoforms [28]. While the IgG glycans display a characteristic signature of partial galactosylation, as well as other features, this signature does vary between individuals and disease states [29].

Mass spectrometric and X-ray crystallographic analyses indicate that glycan pairings at the two Asn297 sites on IgG-Fc can be asymmetric, which further increases the diversity of IgG glycoforms [30], [31]. Overall, IgG N-glycans are predominantly biantennary structures with differences in the degree of fucosylation, sialylated and galactosylation. The majority (~ 90%) of human IgG-Fc glycans are core-fucosylated, out of which a-, mono- and bigalactosylated forms account for ~ 30%, ~ 35% and ~ 16% of the total glycan pool, respectively [32], [33]. About 15% of human IgG-Fc glycans contain a GnT III-mediated bisecting GlcNAc [33], [34]. In serum, a trace amount of bisecting GlcNAc residues are also modified by galactosylation [35]. Only a small proportion of serum IgG Fc glycans are sialylated, with monosialylated and disialylated glycoforms accounting for approximately 5–10% and 1%, respectively [1], [33], [37], [38].

In addition to the conserved glycosylation Asn297 site on IgG Fc, roughly 15–20% of polyclonal human IgGs are glycosylated in the variable regions of the heavy chain, light chain, or both, of the Fab regions [38], [39], [40]. The Fab N-glycans contain more sialic acid, galactose and bisecting GlcNAc residues than Fc glycans [38], [39], [41]. It should be noted that, unlike the N-glycans on IgG Fc, those on IgG-Fab are refractory to release by peptide-N-glycosidase F (PNGase F) [34], [42]. Although the functional significance of IgG Fab glycosylation has not been fully evaluated, results from monoclonal antibodies (mAbs) suggest that glycosylation in the variable regions of the kappa (V_κ_), the lambda (V_λ_) light chains or the heavy chains (V_H_) can have a neutral, positive or negative influence on antigen binding [34].

### 3.2 Cellular production of antibody Fc glycoforms

Analysis of Fc glycoforms by X-ray crystallography and nuclear magnetic resonance (NMR) spectroscopy have shaped our understanding of the factors influencing natural antibody glycosylation and have provided a structural rationale for biophysical and functional effects of glycan engineering [43]. These structural studies have exploited a variety of glycoengineering strategies in the preparation of the target antibody glycoform. These strategies are outlined in Section 4, below.

**Fig. 2:**
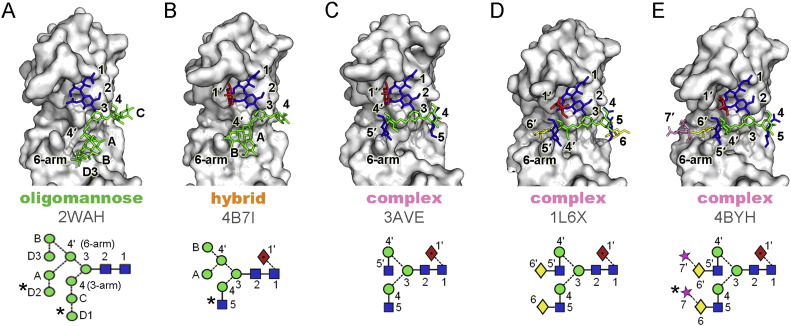
Crystal structures of the glycosylated Cγ2 domain of IgG Fc glycoforms. The following symbols were used to represent glycans [283]: yellow diamonds, galactose; blue squares, GlcNAc; green circles, Man; red diamonds with black dot, fucose; stars, sialic acid. Linkage positions are shown by the angle of the lines linking the sugar residues (vertical line, 2-link; forward slash, 3-link; horizontal line, 4-link; back slash, 6-link). Anomericity is indicated by unbroken lines for β-bonds and broken lines for α-bonds. The system of Vliegenhart et al. [284] is used for labeling residues within oligomannose- and biantennary-type oligosaccharides with the additional modifications of **7** and **7′** for sialic acid, **1′** for α1 → 6-linked core fucose [285]. PDB IDs are displayed in the figure [44], [45], [46], [47], [48], [49]. Residues not evident in the electron density are labelled with an asterisks.

Interestingly, the available crystallographic data almost completely characterizes the structure of the Fc domain throughout the natural biosynthetic stages of glycan remodelling (Fig. 2). Analysis of this panel of structures provides a detailed understanding of the decisive stage in complex-glycan biosynthesis and the generation of a class of thermodynamically more stable complex-type glycoforms [44], [45], [46], [47], [48], [49].

Regardless of the glycoform, the interaction between the protein surface and both the Manβ1–4GlcNAcβ1–4GlcNAc core and what can be observed of the 3-arm, is conserved [44]. In contrast, the 6-arm residues exhibit divergent conformations reflecting the differential chemical compositions and/or surrounding environments of glycan residues. One of the major changes in protein-glycan packing during Fc glycan biogenesis occurs during the transition from hybrid type to complex type. During this transition, Golgi α-mannosidase II processing first causes the relaxation of the 6-arm toward the protein surface. Next, GnT II catalyses the transfer of β1,2-linked GlcNAc to the 6-arm core mannose (Man4ʹ) to form the hydrophobic stacking interactions between GlcNAc5ʹ and Phe243 (Fig. 2). Such glycan-protein stacking interactions have been shown to increase Fc stability while suppressing subsequent enzymatic processing of Fc glycan [44]. This can be further rationalized by considering the impact of such stabilizing interactions on the dynamics of the system. NMR studies utilizing ^13^C labelled terminal galactose residues have shown that approximately half of the glycan population at physiological temperature is highly flexible with the remaining conformation exhibiting relaxation rates concomitant with the protein-bound configuration [50]. In this way, stabilizing interactions drive the population into a conformation that is less accessible to sialyltransferases [51].

**Fig. 3:**
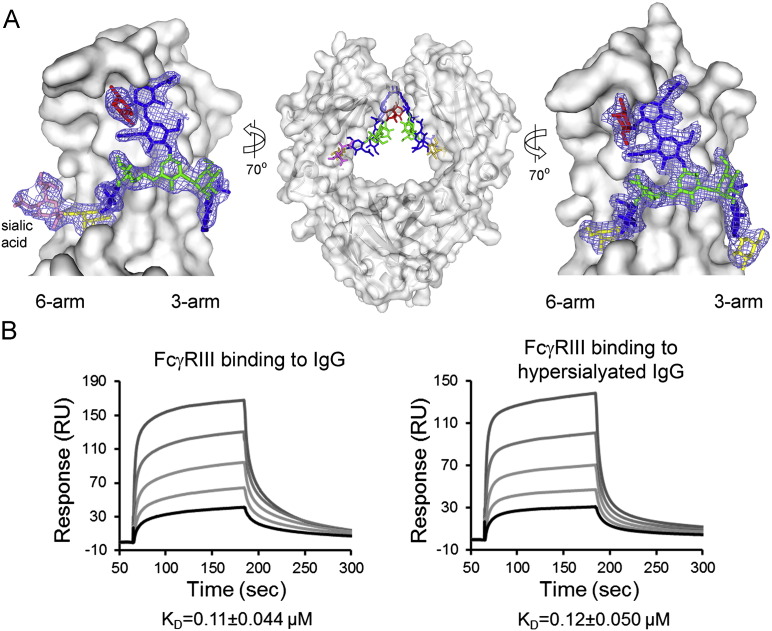
The architecture of α2,6-sialylated IgG Fc and binding to FcγRIIIa is not modulated by Fc α2,6-sialylation. (A) Crystal structure of hypersialylated human IgG1 Fc with 2*F*_o_ − *F*_c_ electron density shown around the N-linked glycans (PDB ID: 4BYH). Panel adapted from Crispin et al. [45]. Glycan residues are coloured according to Fig. 1 (sialic acid, pink). (B) The binding of FcγRIIIa (Val158 variant) to IgG is independent of α2,6-sialylation of IgG Fc as shown by surface plasmon resonance analysis. Panel adapted from Yu et al. [51].

The structure of the sialylated Fc has been extensively debated mainly in the context of understanding the mechanism of the putative anti-inflammatory activity of α2,6-sialylated immunoglobulins. Sondermann et al. hypothesized that α2,6-linked sialylation on the 6-arm would bind to the protein surface driving substantial conformation changes [52], [53]. However, NMR analysis by Barb et al. concludes that “sialylation of either branch terminus does not appear to dramatically alter the motional behavior of the N-glycan as judged by solution NMR spectroscopy” [54]. Furthermore, two independent crystallographic analyses revealed that the 6-arm sialic acid was projecting away from the protein-bound galactose residue in an entirely solvent accessible manner [45], [55] (Fig. 3A). In contrast, those structures suggest that α2,3-sialylation that could occur in recombinant antibody glycosylation would prevent the formation of the canonical galactose-protein interactions with the potential for longer range destabilizing effects through the 6-arm. Evidence for this comes from recent hydrogen-deuterium exchange mass spectrometry (HDXMS) studies which while not examining the naturally occurring α2,6-sialylated glycoforms, presented evidence of destabilisation upon α2,3-sialylation [56]. In addition, the HDXMS analysis confirmed elevated regional solvent accessibility of protein predicted by the crystal structures of the oligomannose and hybrid-type Fc glycoforms [44].

## 4 Emerging strategies for antibody glycoengineering

An important feature of antibody glycosylation is not only the structural heterogeneity and complexity of glycans but also the observation that minor structural changes significantly affect the effector functions of antibodies. Thus, the preparation of homogeneous antibody glycoforms is important both for fundamental structure–function relationship studies and for therapeutic applications. Strategies that allow a greater control of antibody glycosylation profiles mainly involve manipulations of host biosynthetic pathways and/or in vitro chemo-enzymatic glycosylation remodelling [57], [58].

### 4.1 Manipulations of host biosynthetic pathways

#### 4.1.1 Glycoengineering in mammalian cells

Mammalian cells, especially Chinese hamster ovary (CHO) cells, are the predominant system for the production of therapeutic monoclonal antibodies (mAbs). Since afucosylated antibodies have been reported to exhibit enhanced ADCC, a property that is favourable for cancer therapy [59], [60], [61], considerable efforts have been focused on creating mutant cell lines capable of producing non-fucosylated antibodies. One way to achieve this is to knockdown or knockout the *FUT8* gene, which encodes the α1,6-fucosyltransferase (α1,6-FucT) that catalyses the transfer of fucose from GDP-fucose to the innermost GlcNAc residue of the tri-mannosyl core structure [62], [63], [64]. This technology was pioneered by the Japanese company Kyowa Hakko Kirin to create mogamulizumab (Poteligeo®) to treat refractory adult T-cell leukemia [65]. Another approach is to knockout the mammalian *GMD* gene, which encodes the enzyme GDP-mannose 4,6-dehydratase that takes part in the biosynthesis of the fucose donor substrate, GDP-fucose, from GDP-mannose [5], [66], [67]. Since both *FUT8* and *GMD* genes share no redundancy and are independently responsible for α1,6-fucosylation, double knockdown of *FUT8* and *GMD* also effectively allows stable expression of fully non-fucosylated antibodies with enhanced ADCC [68].

Besides the knockout/knockdown mutagenesis, overexpression of certain glycoprocessing enzymes in mammalian host cells can enrich certain glycoforms. For example, overexpression of β1,4-GlcNAc transferase III (GnT III) in CHO cells generates antibodies enriched with a bisecting GlcNAc residue; this addition was subsequently observed to inhibit the fucosylation reaction and thus also results in antibodies with reduced fucose content [69], [70], [71], [72]. This method was the basis of Roche's GlycArt technology, which was employed to create obinutuzumab (Gazyva®) for the treatment of chronic lymphocytic leukemia [73]. Furthermore, since the presence of terminal α2,6-linked sialic acid residues in IgG Fc has been implicated for anti-inflammatory activity [9], [10], [11], [12], overexpression of α2,6-sialyltransferases (α2,6-SiaT) in the mammalian host cell to enhance terminal sialylation has been pursued by some groups [74], [75].

Recently, a method called GlycoDelete has been developed by Meuris et al. to simplify N-glycosylation of recombinant proteins [76]. The group started with an existing GnT I-deficient human embryonic kidney (HEK) cell line, HEK293S, to first ‘trap’ the glycoprotein glycans as Man_5_GlcNAc_2_ [77]. Golgi-targeted expression of an endo-β-*N*-acetylglucosaminidase (ENGase), endoT, in HEK293S cells allows the Man_5_GlcNAc_2_ to be converted to mono-GlcNAc glycoproteins. Subsequent processing by Golgi-resident GalT and SiaT leaves glycoproteins with the galactosylated disaccharides (Gal-GlcNAc) or the sialylated trisaccharides (Neu5Ac-Gal-GlcNAc). In contrast to the many glycan structures produced by wild-type mammalian cells, GlycoDelete cells only produce three variants (mono-GlcNAc, Gal-GlcNAc and Neu5Ac-Gal-GlcNAc) each for the two tested therapeutic glycoproteins, the granulocyte-macrophage colony-stimulating factor (GM-CSF) and an anti-CD20 mAb. Interestingly, both the normally glycosylated anti-CD20 mAb and the GlycoDelete version share similar stability and antigenicity profiles. However, the GlycoDelete antibody has two altered features. First, it has less initial clearance from serum in mice, which might allow a reduced dosage frequency. Second, it has lower binding affinity to human Fcγ receptors. For neutralizing therapeutic antibodies, this feature may improve safety by reducing the risk of cytokine production and immune cell activation. However, for other therapeutic applications that require intact effector functions of antibodies, this feature can be a limitation. Nevertheless, the GlycoDelete technology sets a precedent for the simplification of otherwise heterogeneous complex glycoproteins.

In addition to the knockout/knockdown mutagenesis and overexpression of certain glycoprocessing enzymes, the use of glycoprocessing inhibitors can simplify or redirect glycosylation [78]. For example, deoxynojirimycin and castanospermine inhibit the ER glucosidases I and II to produce the Glc_3_Man_9_GlcNAc_2_ glycoform; deoxymannojirimycin and kifunensine inhibit the ER α-mannosidase to produce the Man_9_GlcNAc_2_ glycoform (Fig. 2A); and swainsonine inhibits the Golgi α-mannosidase II to produce hybrid-based structures such as GlcNAcMan_5_GlcNAc_2_Fuc (Fig. 2B) glycoforms.

### 4.2 Glycoengineering in non-mammalian cells

Non-mammalian cell lines, such as those from yeasts and plants, have been used to generate therapeutic antibodies. Although yeast, plant and mammalian cells exhibit conserved early steps of N-glycosylation, their processing pathways branch off at later points. Yeast glycosylation diverges from humans after Man_8_GlcNAc_2_ is produced in the Golgi apparatus, with the hypermannosylation of the glycan [79]. In plants, after GlcNAcMan_3_GlcNAc_2_ is produced, the core is decorated with bisecting β1,2-xylose and core α1,3-fucose [80].

Strategies to humanize glycoproteins produced in yeast and plant cells mainly focus on: (1) the elimination of the yeast-specific hypermannosylated glycoforms (e.g. by knockout of the *och* or *alg3* genes) or the plant-specific β1,2-xylose and core α1,3-fucose (e.g. by knockout of the β1,2-xylosyltransferase (β1,2-XylT) and α1,3-fucosyltransferase (α1,3-FucT) genes, respectively), and (2) the subsequent transfer of the mammalian glycan processing enzymes (e.g. mannosidases I and II, GnT I and II, β1,4-GalT and α2,6-SiaT) [81], [82], [83], [84], [85], [86], [87], [88], [89], [90], [91], [92], [93]. Examples of successful glycoengineered systems for therapeutic antibodies include the production of the anti-cancer rituximab (Rituxan®) in *Pichia pastoris* [88], the anti-CD30 mAb in *Lemna minor* [90], and the HIV-neutralizing mAb 2G12 and the anti-tumour heteromultimeric IgM PAT-SM6 in *Nicotiana benthamiana* [91], [94].

### 4.3 In vitro chemo-enzymatic glycosylation remodelling

Besides manipulation of host biosynthetic pathways, in vitro chemo-enzymatic glycoengineering is another attractive method to prepare homogeneous glycoforms of mAbs [31], [57], [58], [95]. This chemoenzymatic synthesis comprises three main parts. First, the antibody is deglycosylated by an ENGase, such as the wild type endoglycosidase S (EndoS) from *Streptococcus pyogenes*. This step ensures that the heterogeneous N-linked glycans on the Fc domain are removed, and thus only the innermost GlcNAc or the core-fucosylated GlcNAc is left attached to the Asn residue. Second, selective oxazoline derivatives of complex N-linked glycan structures are prepared by chemical methods using isolated or synthetic oligosaccharides [96], [97], [98], [99]. The third step is transglycosylation, in which the preformed glycan oxazoline donor allows a specific N-glycan to be added to the GlcNAc (or fucosylated GlcNAc) acceptor to achieve a defined, homogeneous antibody glycoform. This transglycosylation is usually catalysed by an ENGase mutant with inactivated hydrolytic activity (e.g. EndoS D233Q, EndoA N171A, EndoA E173Q, EndoM N175A, EndoM N175Q [100], [101], [102], [103]). The chemo-enzymatic approach has been successfully applied to the glycoengineering of human IgG-Fc fragments, as well as various therapeutic mAbs such as rituximab, trastuzumab (Herceptin®) and the antiviral antibody FI6 [100], [104], [105], [106], [107].

## 5 Therapeutic exploitation of antibody glycoforms

### 5.1 Enhancing cell killing activity by defucosylation

Removal of the α1,6-linked core fucose from IgG-Fc glycans has been shown to significantly increase the Fc binding affinity for the activatory FcγRIIIa receptor with enhanced natural killer (NK) cell-mediated ADCC [5], [6], [7], [8], [64], [108], [109], [110], [111] (Fig. 1C). Since ADCC is one of the major mechanisms responsible for the clinical efficacy of mAb in cancer therapies [112], [113], [114], [115], defucosylated antibodies are perceived to be more potent than their fucosylated counterparts. Indeed, this phenomenon has been repeatedly observed in various antigen/antibody combinations, including defucosylated anti-CD19, anti-CD20, anti-Her2, anti-IL-5R and anti-CCR4 antibodies [5], [6], [116], [117], [118], [119], [120]. Although the increase of bisecting GlcNAc residues by overexpression of GnT III from CHO cells was reported to increase ADCC activity [69], [72], this phenomenon was later rationalized by the GnT III-mediated inhibition the α1,6-linked core fucosylation [6]. Thus, it seems that the absence of fucose, but not the presence of this bisecting GlcNAc per se, plays a critical role in ADCC enhancement.

When a non-complexed fucosylated IgG1 Fc fragment was compared with a non-complexed defucosylated counterpart, they were shown to have almost identical structures [121]. However, a crystal structure of the defucosylated Fc-FcγRIIIa complex revealed that the N-glycan attached to the Asn162 of FcγRIIIa interacts directly with the core pentasaccharide of Fc glycan [71], [122], [123] (Fig. 1C). Moreover, NMR data indicated that defucosylation increases the incidence of the active conformation of the Tyr296 of Fc and thus accelerates the formation of a high-affinity complex [123]. Also, thermodynamic data showed that defucosylation enhances binding enthalpy and association rate between IgG1 and FcγRIIIa [7]. In contrast, by steric hindrance, the presence of fucose inhibits positive interactions at the Fc/FcγR interface, while limiting the flexibility of Tyr296 to adapt to an active conformation favourable for the formation of a high-affinity complex [71], [122], [123].

In terms of the molecular and cellular mechanisms, an enhanced affinity between the Fc and FcγRIIIa allows a more efficient activation of FcγRIIIa-bearing NK cells. Both mononuclear leukocytes (NK cells, monocytes, macrophages and γδ T cells) and polymorphonuclear leukocytes (neutrophils, basophils and eosinophils) can mediate ADCC [124], [125]. During the ADCC reaction elicited by defucosylated antibodies, a large number of NK cells, for example, express the activation marker CD69 [110]. The various mechanisms employed to kill target cells have been suggested to include the perforin/granzyme cell death pathway [126], the FAS-ligand pathway [127], the oxidative burst pathway [128], and/or trogocytosis [129], [130].

Furthermore, endogenous serum IgG is known to inhibit therapeutic antibody-induced ADCC [131], [132]. Defucosylated antibodies, due to their higher-affinity to FcγRIIIa, can thus overcome competition from serum IgG for binding to FcγRIIIa on NK cells. Indeed, the inhibitory effect of serum IgG on ADCC was shown to be alleviated by defucosylation of anti-CD20 [8], [133] and anti-Lewis Y [134] antibodies. However, the capacity to circumvent the inhibitory effect of serum IgG is dependent on target density and the serum concentration of the therapeutic antibody that can be achieved [135], [136]. Another potential advantage of defucosylated antibodies is their improved therapeutic activity for all patients independent of the FcγRIIIa polymorphisms. The FcγRIIIa-Val/Phe158 gene polymorphisms, for example, can independently predict response to rituximab in patient with non-Hodgkin lymphoma [112], [113]. Defucosylated rituximab has been shown to reduce the difference between ADCC activities mediated by effector cells expressing low-affinity FcγRIIIa-Phe158 and those by effector cells expressing high-affinity FcγRIIIa-Val158 [137].

Since FcγRIIIa and FcγRIIIb share very high (99%) amino acid sequence homology, the proposed interactions between the FcγRIIIa-attached oligosaccharides and the IgG Fc portion can be extended to the case of Fc-FcγRIIIb interactions. Being selectively expressed in neutrophils but not in NK cells, the FcγRIIIb may have some roles in antibody-dependent cellular phagocytosis (ADCP) [138]. In a study by Shibata-Koyama et al., although defucosylated rituximab did not induce cytotoxicicity when neutrophils were used as effector cells, the antibody did enhance the phagocytosis of dead lymphoma [139].

As a word of caution, it should be noted that besides FcγRIII, other types of Fc receptors like FcγRI and FcγRII are also involved in mediating IgG-dependent ADCC [140], [141], [142]. Thus, depending on the recruited effector cell types, defucosylation may not universally increase ADCC activity. This observation has been reported by Peipp et al., who studied ADCC activities mediated by peripheral blood mononuclear cells (PBMCs) and by polymorphonuclear neutrophils (PMNs) [143]. Although the defucosylated antibody did favour PBMC-mediated ADCC via activation of FcγRIII, its fucosylated counterpart favoured PMN-mediated ADCC via FcγRIIA engagement.

### 5.2 Therapeutic potential of in vivo antibody deglycosylation

Autoimmune disorders refer to the condition in which the immune system has lost its ability to self-tolerate, resulting in the destruction of self-tissue. Accumulating evidence suggests that autoantibodies can contribute to the disease development [144], [145]. For example, arthritogenic antibodies reactive to type II collagen in the cartilage matrix are directly pathogenic even in the absence of inflammatory mediators [146], [147]. Similarly, in pemphigus patients, IgG autoantibodies can directly bind to epidermal cells to disrupt structures that maintain the cell–cell or cell–matrix adhesion in the skin [148], [149]. Alternatively, antibodies as constituents of immune complexes can trigger and maintain chronic inflammation, for instance, via activation of the complement cascade and/or via extensive cross-linking of FcγR-bearing cells. Furthermore, antibodies have been shown to contribute substantially to allograft rejection [150]. Therefore, elimination of such pathogenic antibodies and immune complexes can be beneficial for the patients.

Since deglycosylation impairs the binding of the FcγRs to the antigen–antibody complex and complement activation, this strategy has been increasingly recognized as a targeted treatment of certain autoimmune conditions [151]. For example, immunotherapy that targets the amyloid-β (Aβ) protein has been investigated for Alzheimer's disease [152], [153], [154], [155], [156]. Despite being effective, systemic administration of anti-Aβ antibodies using unmodified IgG can provoke neuroinflammation via microglial activation and thus increases the risk of vascular amyloid and brain microhemorrhage [156], [157], [158]. In contrast, deglycosylated anti-Aβ antibodies greatly reduce such incidents while still retaining the properties of cognition-enhancing and amyloid sequestration [159], [160], [161], [162]. In another study, deglycosylation has been shown to convert pathogenic neuromyelitis optica (NMO)-IgG autoantibodies into therapeutic blocking antibodies [163]. Similar observations have been made in a murine model of fetal alloimmune thrombocytopenia, in which the administration of deglycosylated maternal alloantibodies prevents the endogenous glycosylated autoantibodies from destroying fetal platelets [164].

The therapeutic potential of EndoS to deactivate autoantibodies and alleviate autoimmunity has emerged over the past few years [165], [166], [167], [168], [169]. Similar to many other ENGases, EndoS cleaves the β1-4 linkage between the two GlcNAc residues found in the core of the N-linked glycan of IgG. However, EndoS is highly unusual in its lack of cross-reactivity to other glycoproteins and in its specificity for the complex-type biantennary glycans on serum IgG [95], [170], [171]. As a strategy to evade the host immune system, the bacteria release EndoS, which deglycosylates human IgG molecules and results in impaired FcγR binding and decreased complement activation through the classical pathway [172]. Although such actions increase bacterial survival in human blood [173], EndoS does not seem to be a major virulence factor, at least not during systemic infections [174]. Repeated intravenous administration of EndoS in rabbits has been shown to completely hydrolyse the glycans of the whole IgG pool despite the generation of anti-EndoS antibodies, while causing no adverse effects on the animals [175].

The molecular basis of EndoS deactivation of IgG is of considerable interest. The X-ray crystal structure of EndoS and a model of its encounter complex formed with the IgG1-Fc domain [176], together with the functional analysis of fragments of EndoS and IgG [177], have shed some light on the mechanism. EndoS has been shown to adopt a V-shaped conformation with the glycosidase, leucine-rich repeat (LRR), hybrid Ig, carbohydrate-binding module (CBM) and C-terminal three-helix bundle domains [176]. Molecular modelling of the EndoS/IgG Fc complex suggests that the glycosidase and IgG domains are crucial for the high substrate specificity of EndoS, while the LRR domain is important for the enzyme stability [176]. This model also predicts that glycosylated antibodies enter the V shape and are trapped by the CBM during processing [176]. Several CBM domain residues, namely Trp803, Arg908 and Glu833, have been identified as hot spots for binding by computational alanine scanning mutagenesis [176]. In addition, functional analysis of truncations of EndoS revealed that, although the C-terminus of EndoS is dispensible for activity, its deletion impedes the hydrolysis of IgG glycans [177]. Interestingly, besides full-length IgG molecules, EndoS is also active against different IgG fragments including IgG Fc and the IgG Cγ2 domain (with or without the hinge region) [177]. In the context of *S*. *pyogenes* infection, such activity may further enhance the bacterial immune evasion strategy: EndoS continues to act on IgG fragments produced by other *S*. *pyogenes* proteases such as SpeB and IdeS [178].

Besides being actively explored as a potential therapy for IgG-mediated immune diseases, EndoS can also be utilized to enhance monoclonal antibody (mAb)-receptor interactions [171]. This application exploits the fact that EndoS deactivates IgG molecules with complex-type biantennary glycans rather than those with oligomannose-type glycans. This is in contrast to the related EndoS2, which shows activity to both complex- and oligomannose-type glycans [179]. Thus, upon treatment with EndoS, therapeutic mAbs containing oligomannose-type glycans could overcome FcγR saturation by serum IgG to maintain the FcγR binding activity. Since oligomannose Fc glycoforms fortunately exhibit high-affinity binding to all human FcγRs [180], [181], [182] and since their serum clearance is similar to that of the complex-type glycoforms [183], [184], this ‘receptor refocusing’ strategy can significantly boost the immunological signal provided by therapeutic mAbs. This approach can also be extended to any EndoS-resistant antibodies, including aglycosylated mAbs engineered to exhibit functional FcγR interactions [185].

Taken together, in vivo deglycosylation of antibodies represents a promising platform for the treatment of autoimmune diseases and EndoS-mediated cleavage of IgG opens up avenues for treating inflammation and for enhancing mAb-receptor interactions.

### 5.3 Anti-inflammatory glycoforms and their receptors

Intravenous immunoglobulin (IVIg) preparations are derived from the pooled plasma of thousands of healthy donors. Although IgG is the dominant component, varying amounts of other immunoglobulin isotypes, such as IgA or IgM, may exist in different IVIg preparations [186]. Low-dose IVIg preparations are pro-inflammatory and are mainly used in primary and secondary immunodeficiency disorders. In contrast, high-dose (~ 1–3 g/kg of body weight) IVIg preparations are anti-inflammatory and have been licensed to treat various autoimmune diseases, such as immunothrombocytopenia (ITP) and Guillain-Barré syndrome, and systemic inflammatory conditions, such as chronic inflammatory demyelinating polyneuropathy (CIDP) [187].

Exactly how this anti-inflammatory effect is mediated by IVIg has been an active field of research. Several mechanisms, for which the variable F(abʹ)_2_ and/or the constant Fc regions are accountable, have been proposed and covered by other reviews in depth [188], [189], [190], [191], [192]. In brief, explanations for the F(abʹ)_2_-dependent mechanisms mainly include the blocking of cellular receptors involved in autoimmune pathology, the neutralization of autoantibodies and the inhibition of pro-inflammatory mediators such as C3a or C5a [193], [194], [195], [196], [197]. While such models are attractive, the isolated IVIg Fc-fragment has been shown to be sufficient for anti-inflammation [9], [198], [199], [200], [201], [202]. A role for the Fc is further supported by a requirement for the interactions with certain Fc-binding receptors (FcγRs). They include the activating (especially FcγRIII) and inhibitory (FcγRIIb) members of the classical Fcγ receptors (FcγRs) [203], [204], [205], [206], [207], [208], [209], [210], as well as the neonatal Fc receptor (FcRn) [211], [212]. Other non-traditional inhibitory FcγRs which have been recently implicated are: (a) the sialic acid-binding immunoglobulin-type lectin (SIGLEC) CD22 [213], (b) the C-type lectin receptor dendritic cell-specific intercellular adhesion molecule-3-grabbing non-integrin (DC-SIGN) [199], [214], [215], (c) the C-type lectin dendritic cell immunoreceptor (DCIR) [216], and (d) the Fc receptor-like protein 5 (FCRL5) [217], [218]. Nevertheless, the identity of the receptors involved is still a matter of some controversy [219], [220], [221], [222], [223], [224], [225], [226].

IVIg has been regarded as a pluripotent drug that can work via many different and complex pathways, depending on the types of autoimmune or inflammatory diseases being treated. Here, we would like to focus on the role of sialic acid in the anti-inflammatory activity of IVIg and the potential clinical implications of α2,6-sialylated IVIg (sIVIg) and/or α2,6-sialylated IgG Fc (sFc) therapies.

#### 5.3.1 Anti-inflammatory activity of sialylated IVIg (sIVIg)

The first evidence that the glycan at the IgG Fc Asn297 residue is indispensable for the anti-inflammatory activity of IVIg was provided by Kaneko et al. in 2006 [10]. In contrast to the fully glycosylated IVIg preparation, both of the deglycosylated (PNGase F-treated) and the desialylated (neuraminidase-treated) IVIg forms failed to suppress rheumatoid arthritis (RA) in mice [10]. It was later revealed by Anthony et al. that only the addition of sialic acid residues with α2,6-linkages, but not those with α2,3-linkages, on the IgG Fc N-linked glycans could recapitulate the anti-inflammatory effects of IVIg in vivo [9]. Such a crucial role of IVIg sialylation is also supported by various studies in mice with different autoimmune conditions [11], [12], [214], [215], [227].

Moreover, agalactosylated and asialylated IgG glycoforms have been suggested to be pro-inflammatory due to their associations with various autoimmune and inflammatory diseases [106], [228], [229], [230], [231]. In contrast, a recent study shows that, compared to CIDP patients with stable or worsened conditions, those in remission have significantly increased serum IgG-Fc sialylation [232]. Besides acting as a negative regulator of B cells proliferation independent of FcγRIIb expression [233], [234], Fc-sialylated glycovariants were shown to limit the pro-inflammatory IgG effector functions through impairment of complement-mediated cytotoxicity [232]. Thus, sialylated IgG glycoforms have been hypothesized to act as a switch to restore a balanced immune response. However, which cellular receptor(s) can directly bind to sialylated IgG glycoforms and how such interactions can trigger a downstream anti-inflammatory effect remains an open question. Recent evidence from binding studies suggests that classical FcγRs are unlikely candidates as the affinity of IgG for these receptors either remain unchanged [51] (Fig. 3B) or even decrease upon Fc-sialylation [10], [235].

#### 5.3.2 The receptor(s) for α2,6-sialylated Fc (α2,6-sFc)

One of the proposed candidate receptors for the α2,6-sFc is the C-type lectin receptor DC-SIGN [214]. This notion was derived from the observation that DC-SIGN-transfected CHO cells could deplete only α2,6-sFc, but not α2,3-sFc, from cell culture supernatant [214]. Furthermore, upon sIVIg treatment, DC-SIGN could replace its murine ortholog SIGN-R1 to protect mice from serum-induced arthritis [214], [236]. In this scenario, sIVIg induced the production of IL-33, which subsequently promoted the secretion of T-helper cell type 2 (T_H_2) cytokines, IL-4 and IL-13. Consequently, this led to an upregulation of the inhibitory FcγRIIB expression and a downregulation of the activating FcγRs on myeloid effector cells. In addition, a model for the binding between DC-SIGN and sFc was proposed based on a crystal structure of the CD23/IgE-Fc complex [54]. In this model, non-sialylated IgG-Fc exists in an ‘open’ state with preferential binding to FcγRs, while sialylated IgG-Fc has a ‘closed’ conformation and thus can interact with DC-SIGN.

Although the immunosuppression induced via direct interaction with DC-SIGN has been demonstrated for a number of antigens [237], [238], whether this is true for α2,6-sFc remains debatable. As a type 2 C-type lectin receptor, DC-SIGN contains a carbohydrate recognition domain (CRD) that selectively binds to high-mannose and fucosylated glycans in a Ca^2+^-dependent manner [224], [225], [239], [240]. Yet, none of the current biophysical data indicates a direct interaction between DC-SIGN and α2,6-sFc [223], [224], [225], [239], [240], [241], [242]. Recently, as discussed above, a crystal structure of sialylated IgG Fc [46] has been solved and found to be very similar to that of non-sialylated IgG Fc [243]. Furthermore, no difference was observed for the binding affinity of engineered IgG glycoforms that were either hypersialylated, desialylated or deglycosylated to the extracellular region of the DC-SIGN tetramer [223].

Besides the biophysical data, other immunological studies using human DC-SIGN expressing cells, such as human splenocytes and monocyte-derived dendritic cells (moDCs), did not suggest a clear indication for the involvement of DC-SIGN in the anti-inflammatory activity of IVIg [244], [245], [246], [247]. Although DC-SIGN was implicated in the IVIg-mediated expansion of regulatory T (Treg) cells, this effect was shown to be F(abʹ)_2_- but not Fc-dependent [248]. Also, human DC-SIGN and murine SIGN-R1 differ considerably in terms of their expression and anatomical distribution [249], [250], [251], [252]. For instance, whereas human DC-SIGN is present on subsets of monocytes [236], myeloid dendritic cells (DCs) [253] and subcapsular sinus macrophages [254], [255], murine SIGN-R1 is not expressed on such cell types. Thus, whether DC-SIGN is the proper human homolog of SIGN-R1 requires further investigation.

While a role for DC-SIGN is being re-evaluated, three alternative sIVIg sensors have been proposed so far. They are the sialic acid-binding immunoglobulin-type lectin (SIGLEC) CD22 [213], the C-type lectin dendritic cell immunoreceptor (DCIR) [216], and the Fc receptor-like protein 5 (FCRL5) [217], [218]. Besides being able to bind to N-glycans containing α2,6-linked sialic acid residues with high specificity [256], CD22 was shown to mediate B-cell receptor (BCR) signalling to promote apoptosis in mature human B lymphocytes upon treatment with sIVIg, but not with desialylated IVIg [213]. Arguing against a role for B cells and CD22 is the observation that mice deficient in B cells or CD22 were still protected by IVIg from ITP and serum-induced arthritis [220]. Although this study indicates that IVIg can activate immunosuppressive pathways independently of B cells and CD22, it should not be misinterpreted as direct evidence against the ability of human CD22 to bind human sFc. Instead, it should be understood that murine CD22 may not strongly interact with human sFc.

The most common mammalian sialic acids are *N*-acetylneuraminic acid (Neu5Ac) and *N*-glycolylneuraminic acid (Neu5Gc), which are structurally distinguished by only a single oxygen atom in the C-5 substituent [257]. While mice can synthesize both Neu5Ac and Neu5Gc, humans can only synthesize Neu5Ac due to the non-functional cytidine monophosphate-*N*-acetylneuraminic acid hydroxylase [258], [259]. Yet, while murine CD22 shows a strong preference for Neu5Gc, human CD22 can bind to both Neu5Ac and Neu5Gc equally well [260]. As sIVIg is pooled from human donors and contains Neu5Ac, sIVIg may not interact with murine CD22 sufficiently enough to trigger, for instance, downstream BCR immunosuppressive signalling.

With regards to the remaining two candidates, DCIR and FCRL5, current evidence supporting their involvement in the anti-inflammatory effects of IVIg is limited. DCIR has an extracellular CRD and an intracellular immunoreceptor tyrosine-based inhibitory motif (ITIM) for transduction of negative signals into cells [261]. Mice deficient in DCIR were susceptible to autoimmune diseases at later age due to excessive DC expansion [262]. Previously, it was shown that purified human DCIR could bind to fucose and mannose glycans like LewisB and Man_3_ with high specificity [263], and that such DCIR-glycan interactions could result in signalling via its ITIM motif [264]. Recently, Massoud et al. demonstrated that murine DCIR could specifically bind to sialic-acid enriched IgG (sIgG) in vitro [216]. Moreover, this interaction was shown to be responsible for the expansion of Tregs, which alleviates allergic airways disease in ovalbumin-sensitized mice [216]. Although this is an interesting finding, whether human DCIR can bind to sIgG in a similar manner to murine DCIR requires further examination. Also, whether this DCIR-sIgG interaction is responsible for the anti-inflammatory effects of IVIg in other autoimmune conditions, especially where IgG autoantibodies play a major role, have yet to be explored.

Human FCRL5, together with other Fc receptor-like (FCRL) molecules (FCRL1–6), was discovered when searching for Fc receptor homologs [265], [266]. While the intracellular domain of the classical FcγRs has either an inhibitory ITIM or an activating ITAM motif, FCRL5 has both ITIM and ITAM motifs in its cytoplasmic tail. FCRL5 is preferentially expressed on B cells [267] and is reported to be overexpressed in patients with B-cell malignancies [267], [268], [269], [270]. Recently, it has been demonstarted that FCRL5 is a specific IgG receptor [218], [271]. While the classical FcγRs could interact solely with the Fc region [20], high-affinity binding to FCRL5 required intact full-length IgG molecules [218]. Furthermore, whereas sialic acid enrichment of IVIg using *Sambucus nigra* agglutinin (SNA) enhanced FCRL5-IgG interactions, deglycosylation using PNGase F abrogated nearly all binding activity [218]. Although this study raises the possibility that FCRL5 may act as an sIVIg sensor, whether FCRL5 is indeed the receptor for the α2,6-sFc has not been thoroughly evaluated. For instance, direct comparisons of the binding affinity to FCRL5 between Fc-desialylated (neuraminidase-treated) and Fc-sialylated IVIg samples are not yet available. At the moment, little is known about the immunoregulatory functions of FCRL5 in vivo.

Taken together, while a role for DC-SIGN is not clear, alternative sIVIg sensors, namely CD22, DCIR and FCRL5, have been proposed. More evidence is required to confirm the identity of the receptor(s) for the α2,6-sFc.

#### 5.3.3 To sialylate or not to sialylate?

Despite supporting evidence for the therapeutic applications of sialylated IVIg, there are arguments for sialic acid-independent anti-inflammatory pathways. Indeed, sialylation has been reported to be dispensable in three different in vitro systems [272], [273], [274], as well as in several murine models of ITP [275], [276], experimental autoimmune encephalomyelitis (EAE) [277], arthritis [278] and herpes simplex virus-induced encephalitis [279].

The debate on ‘to sialylate or not to sialylate’ may be further complicated by two main issues. The first issue lies in the different protocols that are used to generate sialylated IgG glycoforms. For instance, polyclonal IVIg purified by SNA lectin chromatography would be predominantly enriched for the F(abʹ)_2_-fragment instead of the Fc [36], [276]. However, whether sialylation of F(abʹ)_2_ reduces the effectiveness of IVIg, as observed by Guhr et al. [276], or whether this enhances the anti-inflammatory activity, as described by Kasermann et al. [272], requires further investigation. Meanwhile, a more effective method to sialylate Fc fragment is the consecutive in vitro enzymatic treatment with β1,4-galactosyltransferase and α2,6-sialyltransferases. Coupled with rigorous industrial-scale protocols and stringent quality control steps, Washburn et al. demonstrated that such enzymatic treatments could successfully generate a tetra-sialylated Fc that was not only devoid of undesirable glycan modifications but also capable of exerting a more potent anti-inflammatory activity than the standard IVIg [11]. The second issue lies in the different dosing protocols for murine models. For instance, in the prophylactic treatment of mice with passive ITP, Schwab et al. administered IVIg 2 h before and counted platelets 4 h after ITP induction [12]. In comparison, Guhr et al. employed a different prophylactic scheme in which mice were given IVIg 24 h before and their platelets were counted 16 h after ITP induction [276]. Variations have also been observed in the murine therapeutic intervention: while Schwab et al. used a constant daily dose of antiplatelet antibodies to induce ITP [12], Leontyev et al. opted for dose-escalation [275]. Of note, while patients receive IVIg upon a confirmed diagnosis, mice in many experiments are administered with IVIg prophylactically. Although a recent study has demonstrated that there is a considerable overlap between prophylactic and therapeutic IVIg treatments in mice with ITP, arthritis and skin-blistering disease [12], it would be more consistent if future murine experiments can be shifted toward therapeutic instead of prophylactic IVIg treatment.

To summarize, although IVIg may activate immunosuppressive pathways independently of sialylation, certain autoimmune conditions could be more effectively treated with sialic acid-enriched IgG glycoforms. Given the extra cost associated with additional enrichment steps for sialylated IgG, recombinant sialylated Fc fragments could be an attractive therapeutic alternative to overcome the current shortage of IVIg [280]. Care needs to be taken when translating the vast knowledge gained on the anti-inflammatory activity of IVIg from murine models to human subjects.

## 6 Concluding remarks

The presence of oligosaccharides attached at a single site on the IgG Fc domain significantly influences antibody effector functions. Recent advances in the field of antibody glycoengineering have provided suitable materials for both fundamental antibody structure–function relationship studies and for therapeutic applications. Manipulation of the biosynthetic pathways of different hosts, including mammalian cells, yeasts and plants, allows the production of selected antibody glycoforms, especially the sialylated and non-fucosylated versions. Alternatively, the preparation of homogeneous IgG glycoforms can be achieved with the in vitro chemo-enzymatic glycosylation remodelling. This method has been successfully demonstrated on various therapeutic monoclonal antibodies such as rituximab and trastuzumab (Herceptin®).

In addition, emerging knowledge on how the Fc glycans contribute to antibody effector functions has opened up new avenues for the exploitation of certain antibody glycoforms in the clinic. For example, defucosylated antibodies have been reported not only to enhance ADCC per se but also to be capable of evading the inhibitory effect of serum IgG on ADCC. While defucosylated antibodies have been increasingly utilized in cancer therapy, especially with the success of obinutuzumab (Gazyva®) for the treatment of chronic lymphocytic leukemia, sialylated versions have been discovered to be capable of suppressing autoantibody-driven inflammation. However, which receptor(s) can interact with the sialylated IgG glycoforms and how such interactions induce the anti-inflammatory activity has been an active field of research.

In conclusion, a better understanding of the structure and function of different antibody glycoforms can provide us with new opportunities to engineer antibody therapeutics with optimal efficacy. Specifically, defining the regulation of IgG glycosylation may represent a promising strategy for fine-tuning IgG-based recombinants for cancer and autoimmune diseases.

## Transparency document

Transparency document Image 1
